# Interfacial Synthesis of Polyaniline/MoS_2_ Nanocomposite Thin Films for Transparent Supercapacitors

**DOI:** 10.1021/acsomega.5c02163

**Published:** 2025-06-02

**Authors:** João Victor Gonçalves, Thauany Hellmann, Amanda F. Pereira, Maria Luiza M. Rocco, Aldo J. G. Zarbin

**Affiliations:** † Department of Chemistry, 355751 Federal University of Paraná (UFPR), CP 19081, Curitiba, PR 81531-990, Brazil; ‡ Institute of Chemistry, 28125 Federal University of Rio de Janeiro (UFRJ), Rio de Janeiro, RJ 21941-909, Brazil

## Abstract

This work describes a strategy to synthesize nanocomposites of polyaniline (PAni) and molybdenum disulfide (MoS_2_) directly as thin films at the interface between immiscible liquids. Based on the interfacial polymerization of aniline in a biphasic acidic aqueous solution/toluene system, four nanocomposites were synthesized by varying the nature and concentration of the acid (HCl or H_2_SO_4_), resulting in films with high homogeneity, transparency, and ease of transfer. The films were characterized by using a range of spectroscopic, microscopic, diffraction, and electrochemical techniques. The results show that a simple modification of the aqueous phase during synthesis led to materials with distinct morphologies, polymer doping levels, and electrochemical properties, as well as different levels of interaction between the components. Furthermore, the films demonstrated potential for application as electrodes in aqueous and transparent supercapacitors, with the highest observed volumetric capacitance exceeding 800 F cm^–3^.

## Graphical abstract

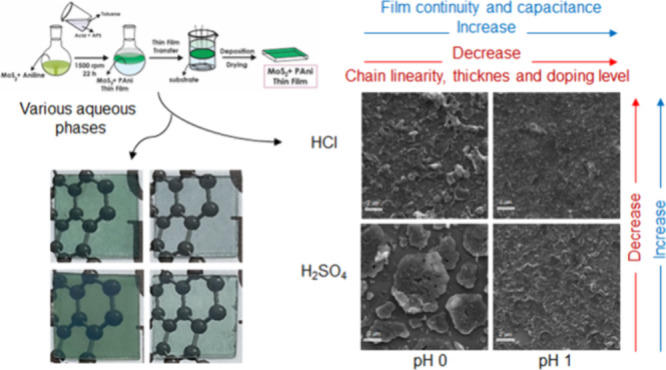

## 1 Introduction

Electrochemical energy storage devices, such as batteries, capacitors, and supercapacitors, are essential for a wide range of applications and efficient energy management. However, further advancements are required to optimize their performance. In particular, the development of aqueous electrolyte-based devices as alternatives to organic electrolyte systems has gained considerable attention due to their safety, low cost, and reduced environmental impact. At the core of this research is the search for materials that share these advantages, enabling the construction of more sustainable and efficient energy storage devices.^1–4^

One widely studied class of such materials is the transition metal dichalcogenides (TMDs). They are a family of layered materials with structures similar to graphite characterized by low toxicity, natural abundance, and unique optical and electrical properties, which are also tunable by controlling the number of stacked layers in a particle.^5–8^ Molybdenum disulfide (MoS_2_) is the most representative member of the TMDs family, in which structure consists of bidimensional layers stacked by weak van der Waals forces, with an interlayer distance of 0.612 nm, where a single layer consists of a plane of molybdenum atoms sandwiched between two planes of sulfur atoms, covalently bonded to the metal centers.^9,10^ The geometry of the S–Mo–S bonds varies, giving rise to three different polytypes of MoS_2_: the metallic 1T-MoS_2_, a metastable phase with an octahedral geometry, and the two semiconducting types, the 2H-MoS_2_ and 3R-MoS_2_ phases, both with a trigonal prismatic geometry. The 2H-MoS_2_ is the thermodynamically stable phase, with a band gap of 1.29 eV, which is dependent on the number of stacked layers, going to 1.9 eV for monolayers.^11,12^

When considering mono- or few-layer MoS_2_, some drawbacks are observed regarding practical applications: the layers naturally tend to restack, affecting the electric properties; there is a natural tendency for partial oxidation in contact with the atmosphere, especially in acidic environments, degrading its structure and altering its properties.^12^ One way to overcome these disadvantages is by preparing composites with conducting polymers, which are interesting due to their flexibility, high environmental stability, and wide range of optical and electrical properties.^13^ Among these polymers, polyaniline (PAni) is the most studied because it is highly stable, inexpensive, easy to synthesize, and can be readily doped by protonation with strong acids.^14,15^ PAni is chemically or electrochemically obtained by the oxidation of aniline, and it occurs in three main oxidation states, which vary in color and conductivity: the yellow and most reduced state, leucoemeraldine; the purple and most oxidized pernigraniline; and the blue and intermediate oxidized state, emeraldine. The nitrogen atoms of those structures can be protonated by acids, resulting in so-called salt structures. Among them, the green colored emeraldine salt (ES) is the most conductive structure of PAni and the charge carriers originated by protonation are known as polarons and bipolarons, which introduce electronic levels within the band gap, responsible for novel electronic transitions associated with this structure.^16^ The degree of protonation of the chains and the nature of the counterions in the structure give rise to various doping states, which translate into a wide range of conductivities, ranging from insulating to semiconducting and metallic characteristics.^17^

Combining PAni and MoS_2_ in a single (nano)­composite material improves their properties and minimizes individual drawbacks for specific applications.^18^ Although the first composites of MoS_2_ and PAni were published by Kanatzidis et al. in the 1990s, their focus was to intercalate polyaniline chains inside the layered structure of bulk MoS_2_, to render the bulk solid electrically conductive.^19^ Their research achieved very interesting results, but it would take until the late 2000s for the first few-layer MoS_2_/PAni composites to be studied. This happened after the isolation of graphene by Geim and Novoselov in 2004, after which they also isolated MoS_2_ and WS_2_ monolayers, highlighting their great potential.^20–22^ Since then, a variety of different composites have been obtained between MoS_2_ and PAni, prepared by solvothermal methods, electrochemical deposition, in situ polymerization, Langmuir–Blodgett process, layer by layer deposition, among others, aiming applications such as capacitors, supercapacitors, electrochemical and biosensors, photovoltaic devices, and more.^23–29^ However, most of these processes obtain the composites as insoluble powders and demand further procedures to process the material in a form suitable for use in systems and devices. Moreover, many of these processes either take several days of synthesis and purification steps or require strict control over the synthesis conditions, such as temperature and pressure.

Processing materials as thin and transparent films is an interesting and useful approach for practical application in several fields because thin films can be transferred and directly employed in the construction of devices. Moreover, it is a very economical approach as it requires only a minimal amount of material.^30^ However, the well-known routes to thin-film processing are not applicable for sophisticated, insoluble, thermally sensitive, and multicomponent materials. In which concerns MoS_2_/PAni composites, there are few reports on processing as thin films, obtained via electrochemical deposition or template-assisted polymerization, but both methods are limited by the nature of the substrate; in electrochemical deposition, the substrate must be conductive, and for template polymerization, the template must be able to withstand the synthesis environment, and the resulting film is limited to the area and form of the template.^18^ Also, there is no control over the interaction between the components.

Our research group has developed a useful way for synthesizing sophisticated materials directly as thin films, the so-called liquid–liquid interfacial route (LLIR), which is an alternative to overcome the aforementioned challenges.^30^ This route is based on utilizing the interface between two immiscible liquids for the preparation and deposition of thin, transparent, and homogeneous films under ambient conditions. Additionally, the LLIR enables control over the nanoarchitecture of nanocomposites by simply tuning the experimental condition, yielding films that are easily transferable to different solid surfaces.^30–37^

The present work reports the LLIR synthesis of MoS_2_/PAni nanocomposite thin films at water–toluene interfaces, exploring four different synthetic conditions by varying the composition of the aqueous phase. The resulting films were characterized by various techniques and compared to neat PAni and MoS_2_ films to investigate the result of the interaction between the components. Finally, the composites were evaluated for their potential application in aqueous and transparent energy storage devices.

## 2 Experimental Section

### 2.1 Materials

Ammonium molybdate ((NH_4_)_6_Mo_7_O_24_·4H_2_O, Vetec), ammonium sulfide ((NH_4_)_2_S aqueous solution 20%, Vetec), sulfuric acid (H_2_SO_4_, 98%, Anidrol), acetonitrile (gradient grade for liquid chromatography, 99.9% LiChrosolv, Merck), hydrochloric acid (HCl 37%, Neon), ammonium persulfate ((NH_4_)_2_S_2_O_8_, ACS Cientfica), and toluene (99.9% Sigma-Aldrich or 99% Neon) were used as received. Aniline (Acros Organics) was doubly distilled under reduced pressure. Molybdenum disulfide (MoS_2_) was chemically synthesized according to our previous report.^31^ The solutions were prepared with deionized water using a Milli-Q ultrapure water purification system with *R* = 18.2 MΩ cm.

### 2.2 Synthesis of MoS_2_/PAni Nanocomposite Films

The nanocomposite thin films were obtained by the LLIR, schematically represented in Figure S1A (Supporting Information): 5 mL of a MoS_2_ dispersion in acetonitrile (0,06 mg mL^–1^) was transferred to a 50 mL round-bottom flask, to which 60 μL of aniline were added, and the flask was sonicated by 30 min (200 W). Afterwards this mixture was put under magnetic stirring at 1500 rpm, and a solution of 38.6 mg of ammonium persulfate dissolved in 30 mL of acidic aqueous solution (HCl or H_2_SO_4_, pH 0 or pH 1), along with 20 mL of toluene, were added to the flask, and the stirring was kept for 22 h. After stopping the stirring, the film spontaneously forms at the interface of toluene/water. The liquid phases were then replaced with fresh ones: the toluene was almost entirely removed using a pipet and replaced with a fresh portion of toluene. The system was stirred for 2 min and then allowed to rest. This process was repeated three times. The same procedure was applied to the aqueous phase, which was subsequently replaced with a pH 3 solution.

### 2.3 MoS_2_ Films

To prepare the neat MoS_2_ film, 5 mL of an acetonitrile dispersion analog of the one mentioned above was transferred to a round-bottom flask and kept under magnetic stirring at 2500 rpm. Then, 30 mL of water and 20 mL of toluene were added to the flask, and the stirring was kept for 22 h (Figure S1B). Again, after stopping the stirring, the film spontaneously formed at the interface, and the liquid phases were replaced in the same way as described before, except that the aqueous phase was replaced by ultrapure water until pH 7.

### 2.4 Synthesis of PAni Films

Neat polyaniline (PAni) films were synthesized and deposited according to the procedure previously reported by our group (Figure S1C).^32^ 38.6 mg of (NH_4_)_2_S_2_O_8_ was dissolved in 30 mL of the acidic aqueous solution. Simultaneously, 60 μL of aniline was dissolved in 20 mL of toluene. Both phases were mixed in a 50 mL round-bottom flask and stirred magnetically at 1500 rpm for 22 h. At the end of this period, a green film was observed at the liquid–liquid interface, with its intensity varying depending on the composition of the aqueous phase. The phases were washed by following the same procedure described for the nanocomposites.

Four different aqueous phases were employed for each of the aforementioned syntheses: aqueous solutions of H_2_SO_4_ or HCl, both at pH 0 (named here as S0 and C0, respectively) or pH 1 (referred here as S1 and C1, respectively). The nomenclature adopted for the 12 samples prepared here is schematized in Table 1.

**Table 1:** Nomenclature of the Samples Prepared in This Work

	**H**_**2**_**SO**_**4**_ pH 0	**HCl** pH 0	**H**_**2**_**SO**_**4**_ pH 1	**HCl** pH 1
neat MoS_2_	M-S0	M-C0	M-S1	M-C1
neat PAni	P-S0	P-C0	P-S1	P-C1
MoS_2_/PAni nanocomposite	MP-S0	MP-C0	MP-S1	MP-C1

### 2.5 Thin-Film Deposition

The films stabilized at the liquid/liquid interface were transferred to the surface of different solid planar substrates (Si, Si/SiO_2_, quartz, glass, 1 × 1 cm). The substrates were fixed in a rod and placed in an empty 100 mL Becker. The entire system (aqueous phase/film/toluene) was transferred to this Becker system and allowed to arrest. The substrate was carefully lifted across the film, which was deposited over its surface. The films were subsequently dried at 60 °C for 24 h. The deposition scheme is represented in Figure S1, and a picture clarifying the deposition of a neat MoS_2_ film is presented in Figure S1d.

### 2.6 Characterization

UV–vis spectra were obtained in a Shimadzu UV2450 spectrophotometer directly on the films deposited over quartz substrates using air as reference. Raman analyses were carried out in WITec Alpha 300 R equipment. All presented spectra are the mean of 1225 spectra collected over an area of 20 × 20 μm of each sample. The excitation line used was 532 nm, with 0.575 mW of power and 3 s of integration time. The samples were comprised of two layers of each film, deposited over a glass substrate. Fourier transform infrared (FT-IR) spectra were obtained in a Fourier transform spectrometer (FT-IR INVENIO-R, Bruker) from 4000 to 600 cm^–1^, with 2 cm^–1^ resolution, at room temperature. Each spectrum was collected from the films deposited over the ZnSe crystal, with 128 accumulations. X-ray diffraction (XRD) was performed using a Shimadzu diffractometer (XRD-6000) with CuKα radiation (λ = 1.5418 Å) and a thin-film accessory. The samples were comprised of two layers of each film, deposited over a glass substrate. The scanning electron microscopy (SEM) images were obtained from the films deposited over Si substrates using a Mira FEG-SEM (Tescan) with an accelerating voltage of 10 kV coupled to an EDS detector (Oxford Instruments) for elemental analysis. The XPS measurements were acquired using an ESCALAB 250Xi spectrometer (Thermo Scientific). Monochromatized Al Kα radiation (1486.6 eV) and a spot diameter of 650 μm were used. The electron energy analyzer operated with a pass energy of 25 eV for high-resolution core-level spectra and 40 eV for survey spectra. The Thermo Scientific Avantage Data System software was used for acquisitions and data processing. A linear combination of Gaussian and Lorentzian functions was employed to generate the fitting curve, and the Shirley function was applied for the background correction of XPS data. Electrochemical measurements were performed with an Autolab potentiostat operated via NOVA 2.1.7 software, using a conventional three-electrode cell with a Pt wire as a counter electrode, Ag/AgCl (3.0 mol L^–1^) as the reference electrode, and the thin films deposited over FTO as the working electrodes. All measurements utilized 0.5 mol L^–1^ of H_2_SO_4_ aqueous solution as electrolyte. Atomic force microscopy (AFM) was used to analyze the thicknesses of the films deposited on the Si/SiO_2_ substrates. A Shimadzu SPM 9700 instrument was used in dynamic mode with an Al-coated NanoWorld NCHR PointProbe Si probe with a force constant of 42 N m^–1^ and a nominal resonance frequency of 320 kHz. A small region of the sample surface was cleaned using 2-propanol to remove a small region of the films for thickness analysis, and the probe scanned the sample surface perpendicular to the interface of the films and the substrate. For each film, the reported thickness is the mean value of 120 points measured across its surface.

## 3 Results and Discussion

Figure S2 shows photographs of the films stabilized at the liquid/liquid interfaces, and the digital images of the films deposited onto planar glass substrates are presented in Figure 1A. As seen in Figure 1A, the four different neat MoS_2_ films are seemingly identical, suggesting that the different aqueous phases do not interfere with the resulting film at the interface. The films of neat PAni, in contrast, have a visible difference in the hue of the green color, characteristic of the emeraldine salt structure of the polymer, which is more intense for the materials prepared at pH 0, and is fainter and more bluish for the ones obtained at pH 1. The composite films look very similar to their respective neat polymer films, suggesting a possible predominance of polyaniline over the optical properties of the composites. The thickness of each film is presented in Table S1, varying from 52 to 95 nm for the neat MoS_2_; from 72 to 400 nm for neat PAni, and from 61 to 379 nm for the nanocomposites. A noticeable variation in thickness is observed for all polymer-containing films, with films prepared at pH 0 and doped with sulfuric acid being thicker. This variance reflects the quantity of film that is obtained from the same amount of precursor material, as can be seen in Figure S2. Overall, all the films obtained present high optical quality, being transparent, continuous, and homogeneous. The transparency of the samples, determined by the transmittance at 550 nm, depends on the composition and thickness of each film, varying from 35 to 78%, as shown in Table S2.

**Figure 1:**
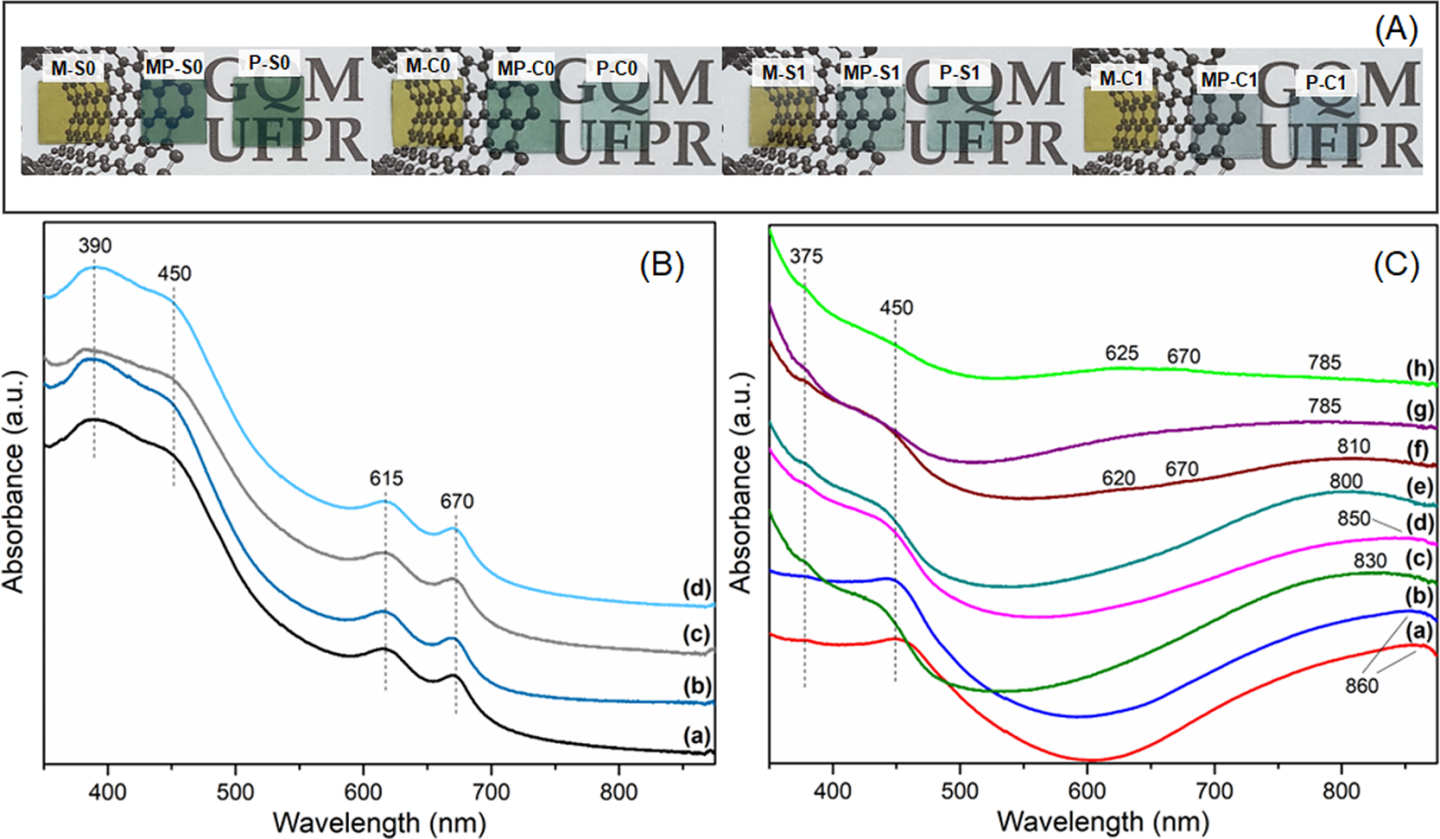
(A) Digital images of the films deposited over glass; (B) UV–Vis spectra of (a) M-S0; (b) M-C0; (c) M-S1; and (d) M-C1; (C) UV–Vis spectra of (a) P-S0; (b) MP-S0; (c) P-C0; (d) MP-C0; (e) P-S1; (f) MP-S1; (g) P-C1; (h) MP-C1.

The UV–vis spectra of the neat MoS_2_ films are presented in Figure 1B, showing all four characteristic bands of MoS_2_: the so-called A and B bands at 670 and 620 nm due to excitonic transitions in the valence band of MoS_2_, related to excitons at the K point of the Brillouin zone; and the C and D bands at 450 and 390 nm due to the transitions between the valence and conduction bands of MoS_2_, respectively.^38,39^ The appearance of these four bands at the same wavelengths and relative intensities indicates that all four MoS_2_ films are essentially analogous.

Figure 1C shows the UV–vis spectra of all of the films containing PAni. The control samples (spectra a, c, e, and g in Figure 1C), exhibit all the characteristics bands of polyaniline in its emeraldine salt structure, at 375 and 450 nm (corresponding to transitions between the valence and conduction bands, and polaronic and conduction bands of the polymer, respectively) and at ∼780–860 nm, due to transitions from the valence band to the polaronic band.^32,40^ This last one presents an energy correlation with the conformation of the polymeric chains, where redshifts indicate more elongated and linear polymer chain conformation, which is also characterized by a predominance of polarons.^32,41^ The differences in this band among various samples provide an initial indication that the experimental conditions employed to prepare the films (composition and concentration of the acids) affect the structure of the obtained polymer: the HCl doped polymer and the samples prepared at pH 1 have less linear chains compared to the H_2_SO_4_ doped and pH 0 ones. Both the effects of dopant and pH variation are most likely related to different growing rates of the polymer in such conditions, since it has been reported that PAni grows more slowly and homogeneously in highly acidic environments and with large dopant anions, such as HSO_4_^–^, and that such factors result in more organized, linear, and/or elongated chains.^42–46^

The spectra of the nanocomposite samples exhibit all the ES bands described previously, but the presence of MoS_2_ appears to promote a higher degree of organization of the polymeric chains, as indicated by the general redshift of the polaronic bands of the nanocomposites (e.g., shifting from 830 to 850 nm for C0 films, and 800 to 810 nm for S1 films); If this interpretation is correct, then the redshift of the composites would also indicate that the MoS_2_ influences the growth rate of PAni, slowing it down. Besides the PAni bands, the characteristic A and B bands of MoS_2_ can also be seen in the spectra of samples prepared at pH 1. The faint signal in these composites, combined with the absence of such bands in the pH 0 films, supports the conclusion that polyaniline optically dominates the composites.

Figure 2A shows the Raman spectra of all of the films containing PAni, in the high-frequency region. The spectra of MoS_2_ are not included, as the samples did not exhibit any bands in this region. Therefore, all the identified bands are characteristic of ES form of polyaniline:^41,47–49^ at 1165 and 1189 cm^–1^, due to the β­(C–H) in benzenoid and quinoid rings; 1218 and 1247 cm^–1^, due to the ν­(N–C) in benzenoid rings and benzene diimine units, respectively; 1317 and 1340 cm^–1^, ν­(C∼N^·+^) characteristic of polaron cation radicals; 1403 cm^–1^, ν­(C–NH^+^), also indicative of formation of polarons; 1467 and 1489 cm^–1^, ν­(C=N−) in deprotonated quinoid units; 1558 cm^–1^, combination of ν­(C–N) in quinoid rings; 1592 cm^–1^, ν­(C=C), also in quinoid rings; and 1636 cm^–1^, ν­(C–C) in benzenoid rings.

**Figure 2:**
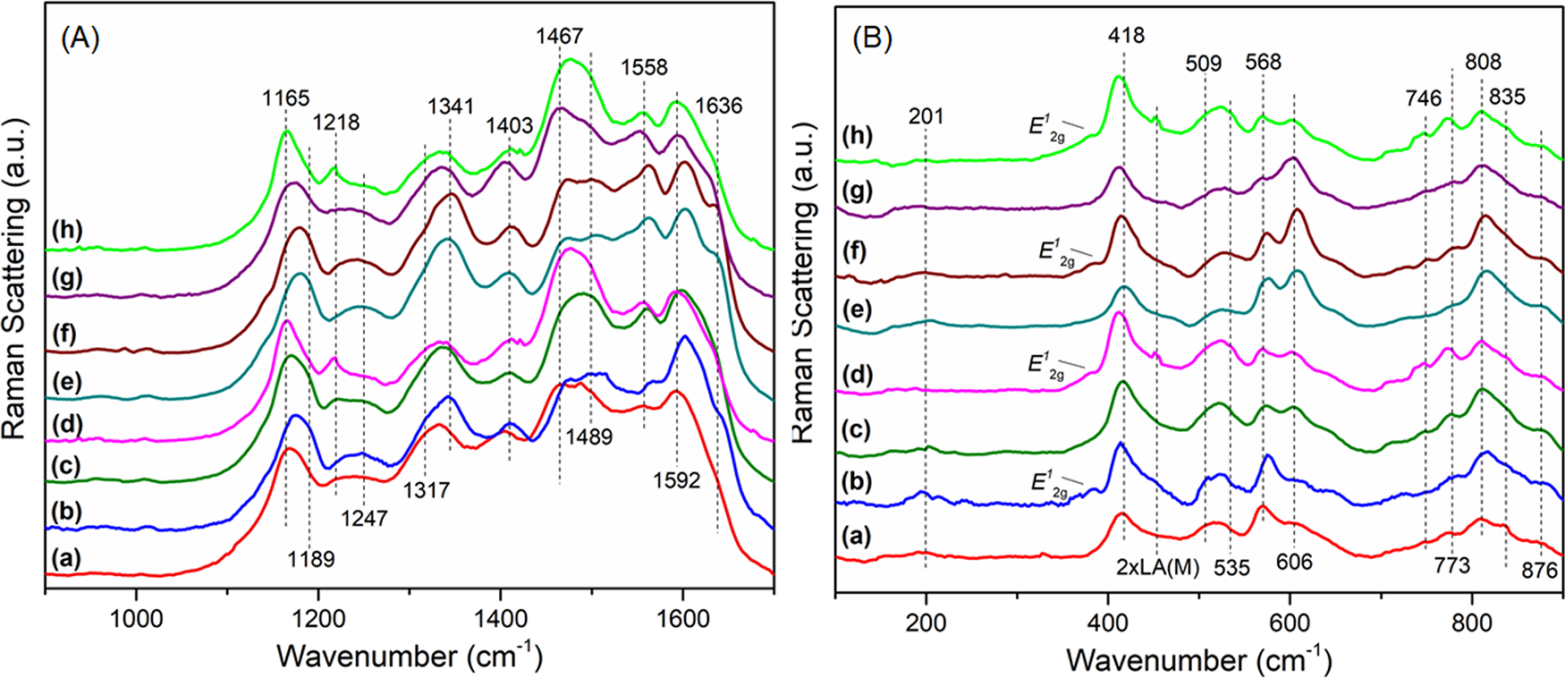
(A) High-frequency and (B) low-frequency Raman spectra of (a) P-S0; (b) MP-S0; (c) P-C0; (d) MP-C0; (e) P-S1; (f) MP-S1; (g) P-C1; and (h) MP-C1.

Analyzing the intensity and position of such ES-based bands, it is noteworthy that the films doped with HCl appear to contain predominantly bipolarons as charge carriers, while the one doped with H_2_SO_4_ shows a combination of both types. This may result from steric effects imposed by the dopant anions, in that HSO_4_^–^, being larger than Cl^–^_,_ could promote charge delocalization, leading to polaron formation to minimize the repulsion between adjacent anions.^43^ Additionally, the higher relative intensity and definition of the bands at 1165 and 1189 cm^–1^ in the composites, along with the ones at 1317 and 1340 cm^–1^, which are also associated with charge carriers in the polymer lattice, suggest that the presence of MoS_2_ improves the doping level of PAni. This is further corroborated by the attenuation of the 1467 and 1489 cm^–1^ modes in the composite films, indicating a higher degree of protonation, in comparison to their neat polymer correspondents. Another relevant band is the one at 1403 cm^–1^, which is known to be linked to polaron delocalization. However, it is also known that this band can be photoinduced by irradiation of the laser during spectra acquisition, which would explain why it exhibits similar intensity across all films.^41^

Further insights can be gained by analyzing the bands at 1247 and 1636 cm^–1^ (related to C–N and C–C stretching in benzenoid rings, respectively) and the ones at 1558 and 1592 cm^–1^ (C–N and C–C stretching in quinoid rings, respectively). Since benzenoid rings are typically associated with polarons—and therefore with more linear polymer chains, and quinoid rings are linked to bipolarons and less linear structures, it can be observed that, in the composites, the bands associated with polarons become more intense and better defined, whereas those corresponding to the bipolarons portions decrease in intensity. The results indicate that polymer chains are more polaronic and linear in the presence of MoS_2_, which aligns with the hypothesis of slower growth and the formation of longer chains, which was already discussed. The dopant and pH of synthesis also influence the carrier distribution: bipolarons appear to dominate in the films doped with HCl and the ones obtained at pH 1, corroborating the data obtained from the UV–vis spectra.

Focusing on the low-frequency region of the Raman spectra, Figure S3 presents the spectra of the neat MoS_2_ films, indicating a predominance of the 2H phase, along with evidence of some 1T-MoS_2_ and molybdenum oxides. The 1T phase is hinted by the presence of the *J*_1_ and *J*_2_ modes, at 147 and 222 cm^–1^ respectively, and the ZA­(A) mode, at 191 cm^–1^, which is exclusive to the 1T phase under the 532 nm excitation line.^11,50^ The other four modes at 286 (*E*_1*g*_), 382 (*E*_2*g*_^1^), 406 (*A*_1*g*_), and 450 cm^–1^ (2xLA­(M)) are common to both phases, but the high intensity of the *E*_2*g*_^1^ and *A*_1*g*_ modes indicates the films are majorly composed of the 2H phase.^11^ Once the initial MoS_2_ sample is composed only by the 2H phase,^31^ the presence of amounts of the 1T structure in the films can be associated with a small phase transition induced by the strong acidic medium of the aqueous phase or by the intensity of the laser beam during the spectra acquisition. The oxidation of MoS_2_ under this acidic condition is well-known and expected, and the occurrence of surface molybdenum oxide is detectable by the very low-intensity bands seen in the Raman spectra presented in Figure S3, in which the attributions are summarized in Table S3.^51–53^

Figure 2B presents the low-frequency Raman spectra of all of the films containing PAni, in which bands related to both polyaniline and MoS_2_ can be identified. The films of neat Pani (control samples) exhibit the following bands: 201 cm^–1^, related to C_ring_–N-C_ring_ angle deformations, indicating the ES-I crystalline form of polyaniline (pseudoorthorhombic cell);^54^ 418 cm^–1^, arising from C–C out-of-plane deformations (16a mode);^54^ 509, 535, and 568 cm^–1^, related to N–C_ring_ in-plane deformations, C–C out-of-plane deformations (16b mode), and cross-linked polymer sections due to the formation of phenazine-like structures, respectively;^49,54^ 746, 773, 808, 836, and 876 cm^–1^, assigned to C–C, N–C_ring_ and C–H quinoid ring, out-of-plane deformations, and quinoid and benzenoid ring in-plane deformations, respectively. These bands are very sensitive to the conformation of the polymeric chains. The high definition of the first three bands indicates few variations on the torsion angles in the chains, which would indicate better organization of the polymer, while the last two represent more planar conformations when more intense and defined.^54,55^ All these bands are detectable in the nanocomposite samples, with some notable differences: (i) the band at 201 cm^–1^ appears to be more intense in comparison to neat polymer films, indicating that the presence of MoS_2_ increases polymer crystallinity, corroborating the data discussed until now; (ii) the band at 418 cm^–1^ presents a blue shift in all the four composites samples, to approximately 415 cm^–1^, which is most probably due to overlapping with the *A*_1*g*_ mode of MoS_2_, that appears at 406 cm^–1^; (iii) the MoS_2_ bands can be observed in the composites spectra, being the *E*_2*g*_^1^ mode at 382 cm^–1^, and the 2 × LA­(M) mode at 450 cm^–1^;^11,50^ (iv) the three vibrational modes at 509, 535, and 568 cm^–1^ become more intense and defined in the composite films (especially in MP-S0), when compared to the neat polymers, which indicates reduced torsional angles in the polymeric chains, indicating again that the MoS_2_ stabilizes a more planar conformation of PAni, while also promoting a higher interaction between chains by cross-linking. The bands at 746, 773, 808, 836, and 876 cm^–1^ also appear to be more defined, and the last two more intense, in the composites, which points again to increased planarity and organization of the chains in the presence of MoS_2_. This could be due to the slower growth of chains in such conditions, but it could also be explained if the MoS_2_ flakes act as seeds or templates for polymer growth, at least during the initiation of chain propagation. Moreover, this increase in organization would also justify the higher doping levels of the polymer in the composites, since better-sorted chains are more easily protonated.

The information obtained by Raman spectroscopy can be further corroborated by FT-IR analysis, presented and discussed in the SI (Figure S4).

Figure S5 shows the X-ray diffractometry profiles of the films. The peak related to the (002) planes of the 2H phase of MoS_2_ can be seen in the profiles of all neat MoS_2_ and composite films.^21^ Some PAni-based films also show the low-intensity peaks attributed to the (110) and (111) planes of the pseudoorthorhombic configuration of PAni,^56^ corroborating the data obtained by Raman spectroscopy.

Figure 3 shows the SEM images of all samples. The morphology of all neat MoS_2_ films is the same, and in accordance with MoS_2_ thin films reported by us,^31^ characterized by a continuous network of small flakes of MoS_2_ interconnected by their borders. Otherwise, the morphology of PAni-containing samples varies significantly under the different experimental conditions. In general, both neat PAni and the composites consist of agglomerates of polyaniline fibers, creating plate-like structures seen in the images presented in Figure 3.^18,32^ However, the shape and size of such plates and the connectivity of these plates are very different and can be related to variations in the growth process of the polymer, as mentioned before. For the S0 films, the PAni structure is very compact and has a more pronounced three-dimensional character when compared to the other samples. These films are also the most discontinuous, as can be evidenced by the exposed substrate between polymeric domains. Such an organization is compatible with a slower growing process and longer, more planar chains because these properties promote better interaction between chains. Also, the polymer in this condition appeared to display a higher degree of cross-linking of the chains, as evidenced by Raman spectroscopy, which would also contribute to a more compact structure. In the composite film, the MoS_2_ flakes can be seen decorating the entire surface of the polymer plates, indicating a good interaction between the two components. The C0 films, while still being somewhat compact, display polymeric plates that are a little more spread out, which contributes to more continuous morphology of the film, indicating the growing process was slightly faster them the previous, as can be expected, since the counterion plays an important role in the growth rate of PAni, with smaller anions such as Cl^–^ promoting faster chain propagation. For the composite in this condition, MoS_2_ is also observed decorating polymer plates, despite some areas displaying bare plates, devoid of MoS_2_ flakes. This probably happens due to a decrease in film thickness, when compared to the S0 ones, which causes the film to spread across a larger area, as seen in Figure S2, discussed previously. Finally, the morphology of the polymer in samples S1 and C1 is very similar, composed of larger, more bidimensional plates, which indicate faster growth and less aggregation of the PAni fibers, probably due to the smaller and less linear chains. In terms of continuity, these films are more continuous than their pH 0 counterparts, with C1 being the most continuous of all and with the largest observed polymer plates. The distribution of MoS_2_ in the composites in these conditions becomes even more precarious than the C0 case, with small agglomerates of the material surrounded by the large and dispersed plates of PAni. This can again be explained by their relative concentration, as the pH 1 films are less than half as thick as their pH 0 counterparts, causing a larger spread of the MoS_2_. Also, this difference in distribution of MoS_2_ provides an explanation for the fact that its excitonic bands are only observable at the pH 1 composites in the UV–vis spectra, because the entire area of the film is saturated by polymer for the pH 0 ones, causing the MoS_2_ bands to be masked by the PAni bands, while the pH 1 composites present these isolated MoS_2_ spots, allowing its excitonic bands to remain optically accessible.

**Figure 3:**
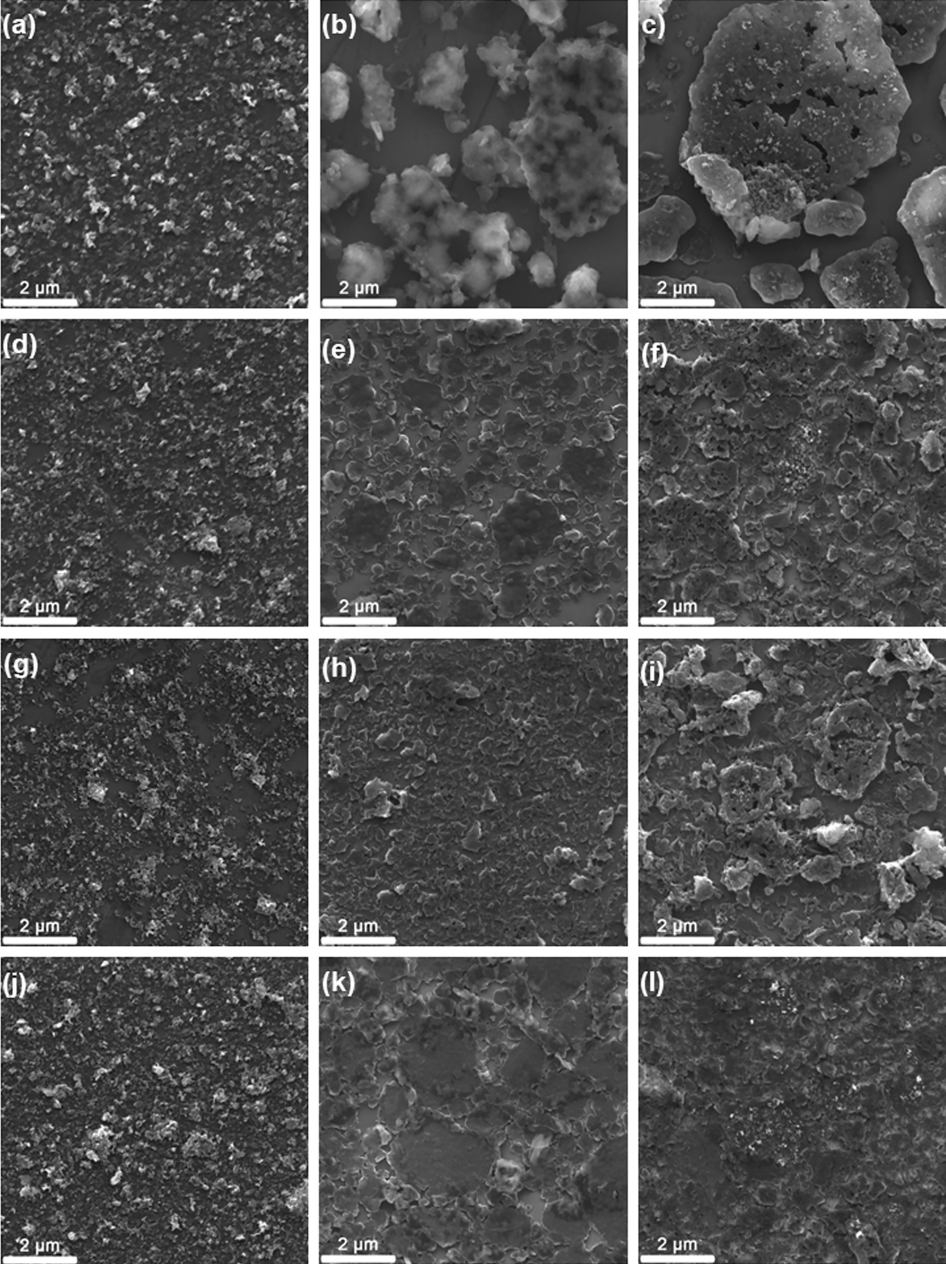
SEM images of (a) M-S0; (b) P-S0; (c) MP-S0; (d) M-C0; (e) P-C0; (f) MP-C0; (g) M-S1; (h) P-S1; (i) MP-S1; (j) M-C1; (k) P-C1; and (l) MP-C1.

The XPS survey spectra (Figure S6) of the films show all the expected elements, as well as contributions from the substrate: the corresponding signals of C 1s, N 1s, S 2p, Mo 3d, and S 3d, confirming the formation of the nanocomposites.

High-resolution core-level spectra of carbon, nitrogen, molybdenum, and sulfur were also measured. The high-resolution N 1s spectra of the nanocomposites (Figure 4) and of the pure PAni (Figure S7) are characterized by four contributions. The peak at lower binding energy (398.5 eV) is attributed to imine (=N−), while the peak centered at 399.5 eV is related to the presence of amines (−NH−). The other contributions at 400.4 and 401.7 eV are assigned to cationic species.^57,58^

**Figure 4:**
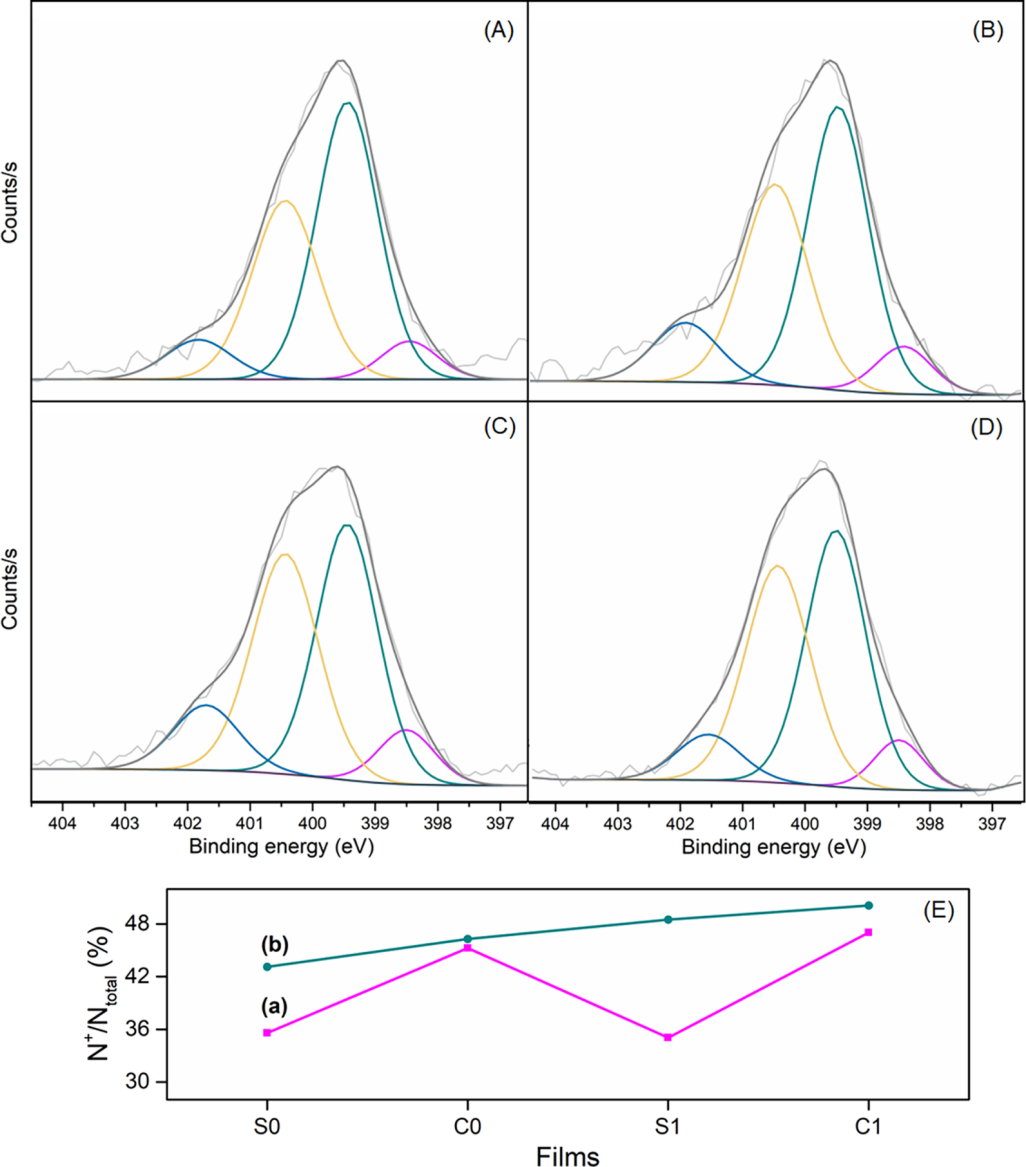
N 1s core-level spectra for (A) MP-S0; (B) MP-C0; (C) MP-S1; and (D) MP-C1. (E) Level of doping (%) of (a) neat PAni and (b) nanocomposite films.

The ratio between positively charged species and total nitrogen enables assessment of the doping level of the polymer. Thus, as shown in Figure 4E, both neat polyaniline and the nanocomposites exhibit higher doping levels for films that were produced with HCl solution. This result is consistent with previous observations, where the level of doping depends on the nature of the dopant acid, and the higher degree of protonation of PAni has been observed as a result of a stronger dopant acid.^59–62^ Moreover, the nanocomposite samples display increased doping levels compared to the neat polymer films, suggesting that the presence of MoS_2_ enhances the doping efficiency, as evidenced by the Raman results. This enhancement in doping can be attributed to the increased organization of the polymer chains promoted by MoS_2_, which facilitates protonation, thereby increasing the concentration of charge carriers in the material.

In the high-resolution S 2p spectra (Figure 5), a low-binding-energy doublet centered at 162 eV corresponds to 2H-MoS_2_.^11,63^ Additionally, a second contribution near 168 eV is present in all nanocomposites, attributed to S­(VI) (2p_3/2_) and associated with the counterions (HSO_4_^–^) in the samples prepared with H_2_SO_4_. The presence of these signals in all samples suggests that they also originate from residual species from the synthesis, particularly from the ammonium persulfate oxidant used during polymerization, whose reduction leads to the formation of sulfate-containing species. In the MP-C0 films, a peak at around 164 eV is also detected. This contribution can be attributed to the C–S bond, indicating a strong interaction between polyaniline and MoS_2_ in the nanocomposites. Notably, this signal is absent in the MoS_2_ reference samples (Figure S8). This C–S bond may facilitate more efficient ion and electron transport, thus enhancing the electrical and structural performance of the nanocomposites.^64–66^

**Figure 5:**
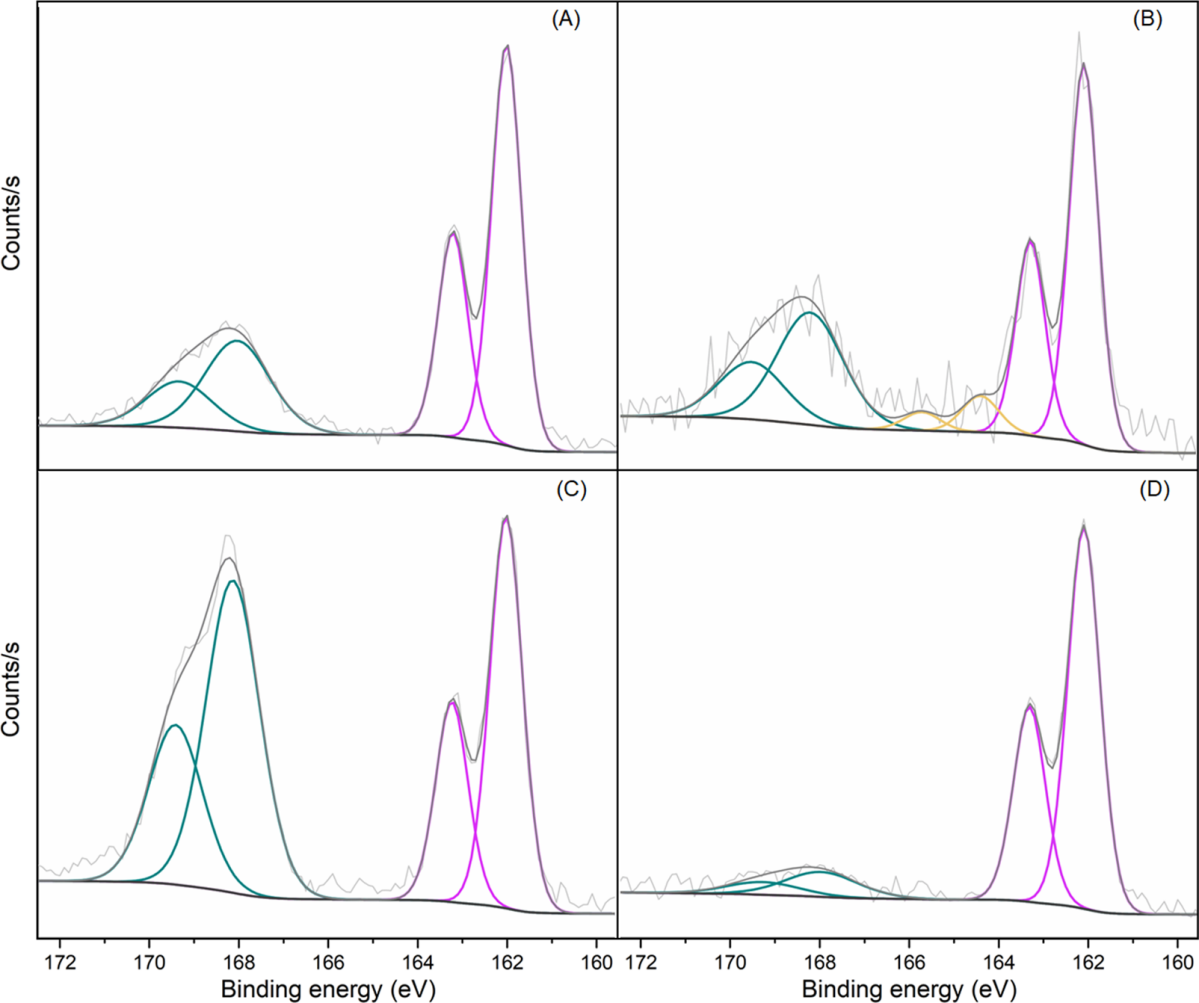
S 2p core-level spectra for (A) MP-S0; (B) MP-C0; (C) MP-S1; and (D) MP-C1 films.

The high-resolution Mo 3d spectra (Figure 6 and Figure S9) are characterized by the presence of a singlet at approximately 226.5 eV attributed to S 2s and a doublet, with the Mo 3d_5/2_ peak centered at 229.3 eV assigned to Mo (IV) in 2H-MoS_2_. Additionally, two further doublets are observed at higher binding energies, with Mo 3d_5/2_ peaks around 231.4 and 233.1 eV attributed to Mo­(V) and Mo­(VI), respectively. The Mo­(VI) component is associated with MoO_3_, indicating partial oxidation, corroborating the data obtained by Raman spectroscopy. Meanwhile, the presence of Mo­(V) can be attributed to the partial degradation of MoO_3_ upon exposure to X-rays.^67^

**Figure 6:**
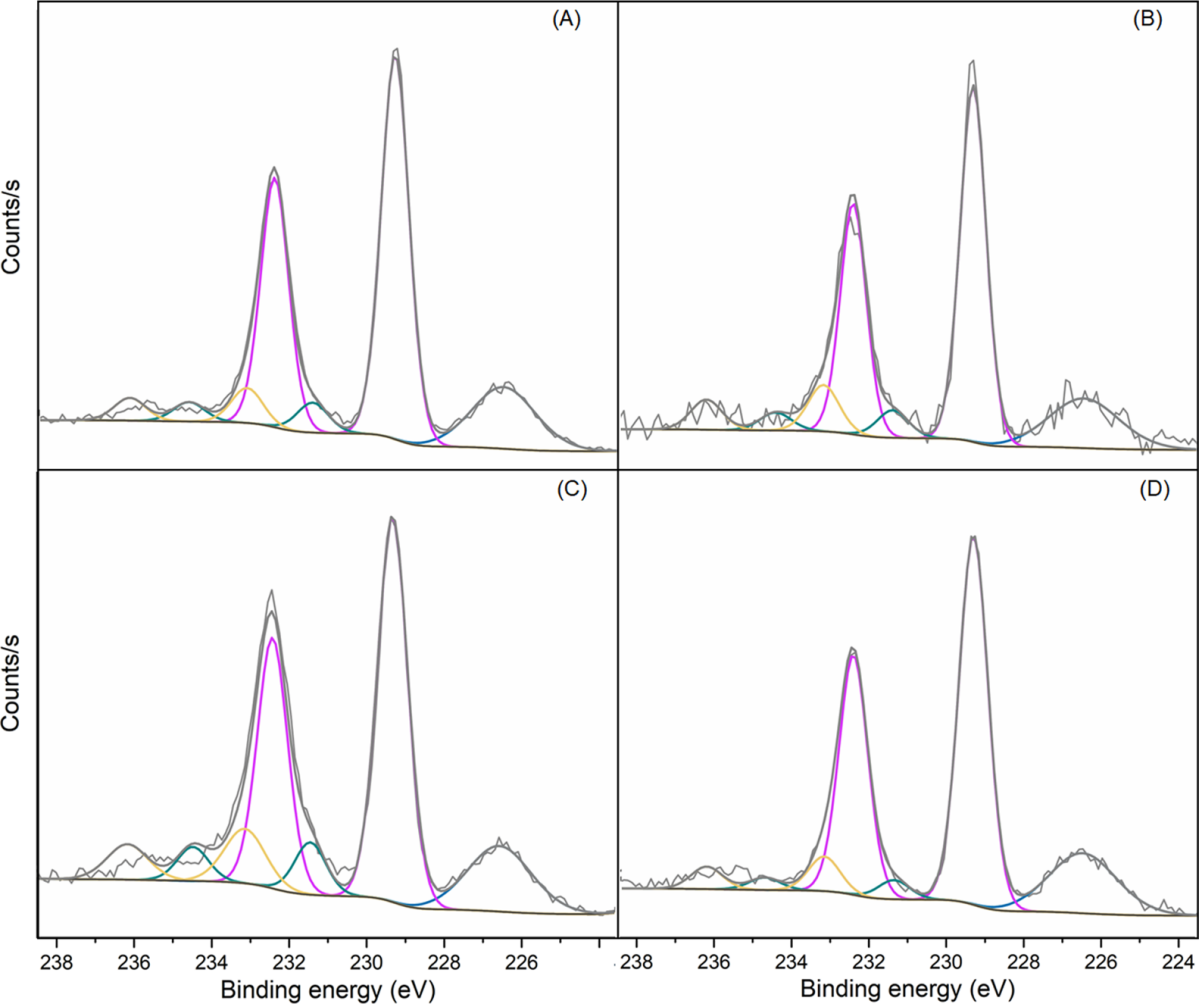
Mo 3d core-level spectra for (A) MP-S0; (B) MP-C0; (C) MP-S1; and (D) MP-C1 films.

To evaluate the electrochemical behavior of the synthesized films, cyclic voltammetry (CV) and galvanostatic charge–discharge (GCD) analyses were performed in a H_2_SO_4_ 0.5 mol L^–1^ aqueous electrolyte. Figure 7A–C shows the resulting voltammograms recorded at a scan rate of 5 mV s^–1^. For the neat MoS_2_ films, two redox pairs can be faintly observed at *E*_1/2_ 0.21 and 0.48 V. Both processes are reported to be from proton intercalation/insertion in the interlamellar space of the MoS_2_ structure, upon partial reduction of exposed Mo­(IV) sites.^68,69^ It has also been observed that the first redox pair is generally observable only when the electroactive MoS_2_ is comprised of at least a small portion of the 1T phase of this material.^68^

**Figure 7:**
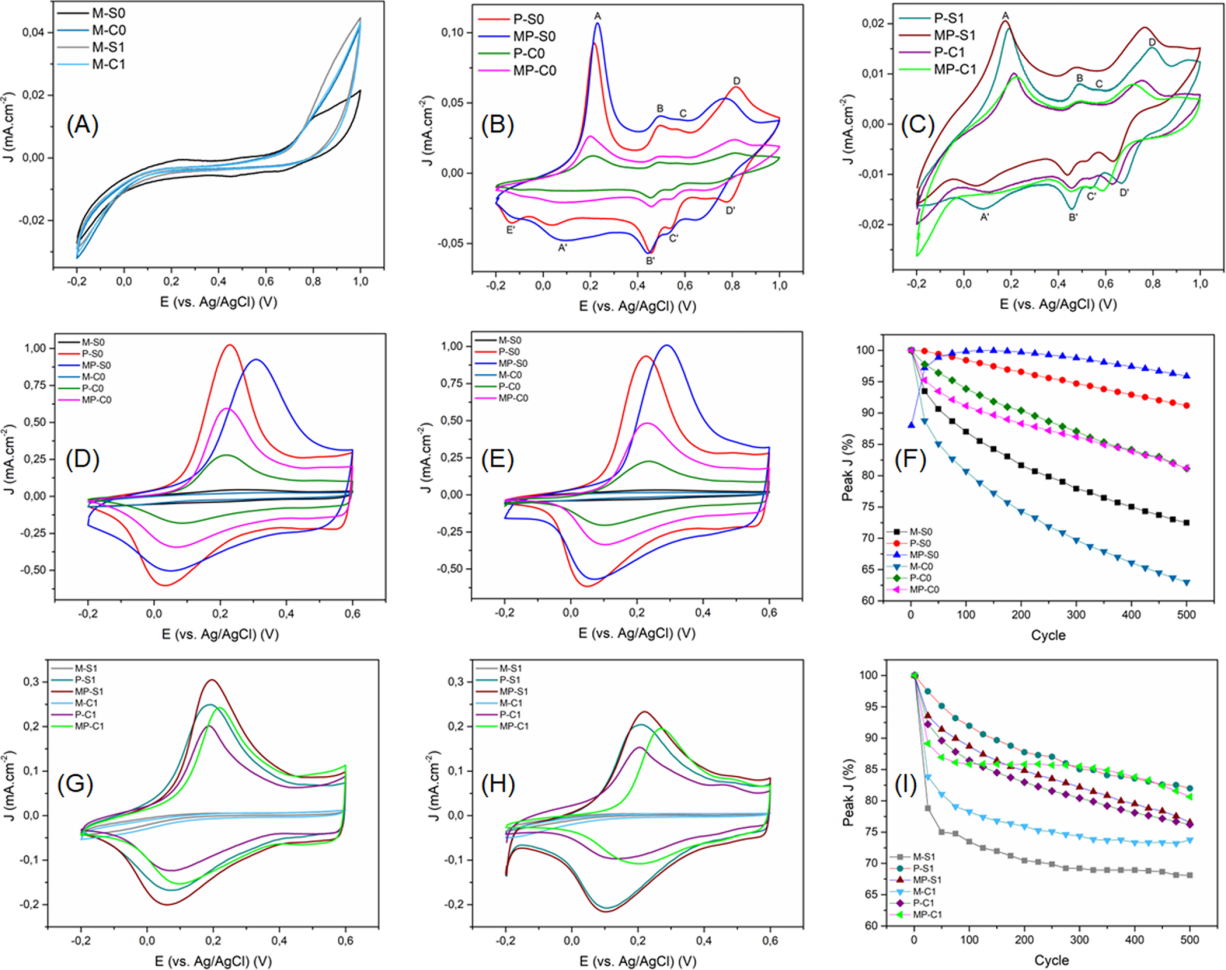
(A–C) Cyclic voltammograms of all films, at scan rate 5 mV s^–1^, between −0.2 and 1.0 V; (D, G) First, and (E, H) 500th voltametric cycle of all films, with scan rate 50 mV s^–1^, from −0.2 to 0.6 V; (F, I) evolution of peak current density over 500 cycles, of the process A for PAni-containing films, and the process at *E*_1/2_ 0.48 V for neat MoS_2_.

The voltammograms of the PAni-containing films are dominated by the characteristic redox processes of the polymer. The peak pair A/A′, at ∼*E*_1/2_ 0.1 V, is assigned to the oxidation of leucoemeraldine to emeraldine, while the D/D′ pair at ∼*E*_1/2_ 0.8 V corresponds to the transition from emeraldine to pernigraniline.^65^ The two smaller pairs in the middle, B/B′ and C/C′, at ∼*E*_1/2_ 0.48 and 0.56 V, respectively, are attributed to side reactions such as polymer cross-linking or degradation, as well as the minor feature labeled E′.^14^ Given the small intensity of the MoS_2_-related processes, their contribution to the voltammetric profiles of the composite films is likely obscured by overlap with the dominant PAni redox processes.

Comparing the voltammograms, the A/A′ and D/D′ redox pairs are generally more defined in the composites, along with a slight shift toward lower potentials, suggesting that both phase transitions are facilitated in the composites, which could be explained by the higher organization and interaction levels of PAni chains in these films, as evidenced earlier. These structural features enhance charge transport and electron delocalization, thereby favoring the redox transitions.^32,70^ Another noticeable effect in the composites is the reduced intensity and definition of the two redox pairs in the middle, indicating a less-cross-linked and more stable polymer obtained in the presence of MoS_2_. Finally, the increased area under the composite voltammograms, when compared to the neat polymers, shows that the combination of the components increases the capacitive current of the films.

The electrochemical stability of all films was tested by performing 500 consecutive voltametric cycles in the potential window from −0.2 to 0.6 V, and the results can be seen in Figure 7D–I. The films containing PAni display a significant shift of the A peak to higher potentials, after 500 cycles, indicating that the leucoemeraldine-to-emeraldine phase transition becomes less favorable after many cycles. This could be due to some reticulation/degradation of the chain during the cycling, as evidenced by the emergence of the B peak after the 500th cycle. It is noticeable that such an effect is less pronounced for the composite films, with the B peak intensity being lower and the potential variance of the A peak smaller, suggesting that MoS_2_ helps suppress degradation and stabilizes the redox behavior of PAni. Moreover, the B peak is more prominent in the pH 1 films, indicating that these are more susceptible to reticulation, in comparison with the pH 0 films, which makes sense since the structure of the latter is more compact and organized, making it harder for reticulation to occur. Figure 7F,I presents the relative variation of the A peak current density over 500 cycles (for neat MoS_2_, the evaluated peak was the one at *E*_1/2_ 0.48 V) normalized to the maximum current density observed. All polymer-containing films maintain a current density retention above 75% after 500 cycles, with the neat MoS_2_ presenting a lower retention, in the range of 60–75%. These values are within or very close to the retention values reported in the literature for similar systems, which usually range from 85 to 95%.^13,32,71–74^ Furthermore, the composites are again evidenced to be more stable in comparison to their respective neat polymers, showing greater retention.

Galvanostatic charge–discharge analyses were employed to evaluate the potential application of the composites as electrodes in energy storage devices. For that, the applied current was normalized by the volume of each film, calculated from the working electrode area and the film thickness. Figure 8A,C displays the GCD curves for all films at a current density of 2.5 A cm^–3^, in a H_2_SO_4_ 0.5 mol L^–1^ aqueous electrolyte, within the potential range of 0.0–0.6 V. All curves present the near-triangular shape characteristic to supercapacitors, with the polymer-containing films displaying a small plateau around 0.15 V during both charge and discharge, related to the faradaic process of PAni in this region.^75^ It can be seen that the neat MoS_2_ films present the fastest charge–discharge time, indicating the lowest capacitance. Following that, the neat PAni films present the intermediate results, with longer charge–discharge times but still shorter than those of the composites, suggesting higher apparent capacitance. Also, the aforementioned plateau is more defined in the composite curves, showing once more that the MoS_2_ facilitates the redox process of PAni, enhancing the pseudocapacitive contribution.

**Figure 8:**
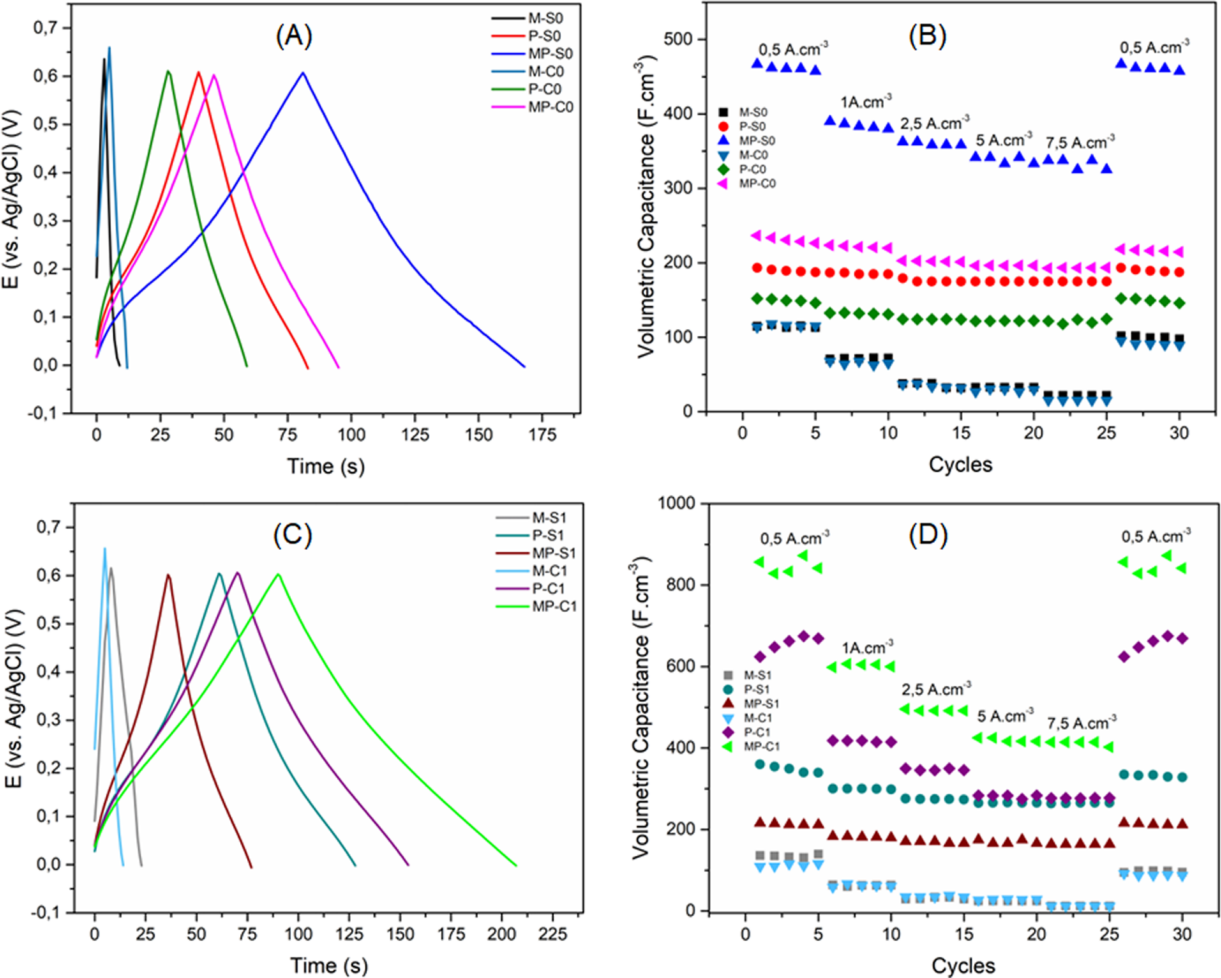
(A, C) GCD curves for all films, at applied current density of 2.5 A cm^–3^; (B, D) volumetric capacitance vs CD cycles with varying applied current densities.

From the GCD curves, along with additional curves obtained at the same and different current densities, the volumetric capacitances of the films were calculated through eq 1:

$$C_{v}=\frac{\left(t\times i\right)}{\left(\Delta E\times v\right)}=\frac{t}{\Delta E}\times J$$ 1

where *C*_*v*_ is the volumetric capacitance (F cm^–3^), *t* is the discharge time (s), Δ*E* is the potential window (V), and *J* is the volumetric current density (A cm^–3^).^76^ Each current density was measured five times consecutively before increasing to the next value, resulting in the graphs displayed in Figure 8B,D. From those, the volumetric capacitance decreases slightly with increasing current density, as expected, but retains approximately its initial value even after being cycled through the highest current density. Calculated volumetric capacitances of all films across all current densities applied are summarized in Table 2. Overall, the composites do in fact have the highest volumetric capacitances, with the two best results being MP-C1, followed by MP-S0. Compared to their neat polymer counterparts, these two composites exhibit overall increases in C*v* of approximately 40 and 100%, respectively. When compared to neat MoS_2_, the increase ranges from 300 to 600%, clearly demonstrating the synergistic effect of combining the two materials. The volumetric capacitances achieved, especially for MP-C1 and MP-S0, are very promising and fall within the range reported for similar systems, as displayed in Table S4, highlighting their potential for application in aqueous supercapacitor devices.

**Table 2:** Mean Volumetric Capacitances (F cm^–3^) for All Films, across the Different Applied Current Densities

sample	0.5 A cm^–3^	1 A cm^–3^	2.5 A cm^–3^	5 A cm^–3^	7.5 A cm^–3^	0.5 A cm^–3^ (final)
M-S0	114	71	35	32	21	100
M-C0	115	65	34	28	15	91
M-S1	135	62	31	24	12	97
M-C1	112	62	35	28	12	88
P-S0	189	185	175	175	175	189
P-C0	149	132	124	121	121	149
P-S1	348	299	274	265	265	331
P-C1	655	417	347	281	277	651
MP-S0	461	384	360	338	332	459
MP-C0	231	221	202	196	193	216
MP-S1	213	182	169	170	164	213
MP-C1	846	603	492	420	412	845

The explanation of why MP-C1 and MP-S0 showed the best results is most likely attributed to the high cohesion observed in the morphology of these films, since it is reported that the capacitance of polyaniline is highly dependent on this property, with cohesive organizations giving better results than the ones with some variation in morphology across the material.^18^ With that in mind, the SEM images would indeed indicate that these two films are the most cohesive ones, with MP-S0 being highly compact and consistent in MoS_2_ distribution and MP-C1 being the most continuous films, with polymer plates showing only slight variations in area, while the other two composites display more pronounced heterogeneity in their polymer structures. Furthermore, the better result of MP-C1 is most probably related to its reduced thickness and higher surface area, which facilitate the contact and penetration of electrolyte into the polymer lattice, enhancing the surface charge accumulation. In contrast, the thick and compact structure of MP-S0 may restrict electrolyte mobility through the polymer lattice, thereby excluding the more internal chains from participating in charge storage, resulting in lower capacitance.

## 4 Conclusions

MoS_2_/PAni-based nanocomposite films were synthesized by using the LLIR method, employing different acids and pH values, resulting in thin, homogeneous, and transparent films that can be easily deposited onto a variety of substrates. The results show that both the type of acid and the pH significantly affect the morphological, electronic, and electrochemical properties. The characterization techniques employed revealed that the composites exhibit longer and more organized polymer chains, which in turn contribute to higher doping levels and more efficient redox processes. All these effects have a strong influence over the performance of the films as electrodes for aqueous supercapacitors, which becomes evident by the substantial increase in volumetric capacitance that the nanocomposites showed, in comparison to their respective neat components. These results were remarkable, especially considering the highest volumetric capacitance observed of 846 F cm^–3^, which is a great value for composites like these, highlighting their potential as electrodes for aqueous supercapacitor devices.

## Supplementary Material



## Notes

Experimental schematics for obtaining MoS_2_, PAni, and MoS_2_/PAni films, including film thickness data, digital images of the films at the liquid–liquid interface, additional spectroscopic data and discussion for all samples, and comparison of the volumetric capacitances observed with results from the literature (PDF)
