# Supplementary material for: Interfacial Synthesis of Polyaniline/MoS2 Nanocomposite Thin Films for Transparent Supercapacitors
Source: ACS Omega. 2025 Jun 2;10(22):23514–27. doi: 10.1021/acsomega.5c02163 (PMC12163641; doi:10.1021/acsomega.5c02163)
Supplement: Supplementary file 1 [file ao5c02163_si_001.pdf]

SUPPORTING INFORMATION

Interfacial Synthesis of Polyaniline/MoS_2 Nanocomposite Thin Films for Transparent Supercapacitors

João Victor Gonçalves,[†] *Thauany Hellmann,*[‡] *Amanda F. Pereira,*[†] *Maria Luiza M. Rocco,*[‡] *Aldo J. G. Zarbin**[†]

[†] Department of Chemistry, Federal University of Paraná (UFPR), CP 19081, 81531-990, Curitiba, PR, Brazil

[‡] Institute of Chemistry, Federal University of Rio de Janeiro (UFRJ), 21941-909, Rio de Janeiro-RJ, Brazil

*Corresponding author.

E-mail address: aldozarbin@ufpr.br

Phone number: +55 41 3361-1565

Postal address: Department of Chemistry, Federal University of Paraná (UFPR), Centro Politécnico, CP 19032, ZIP code: 81531-980, Curitiba, PR, Brazil.

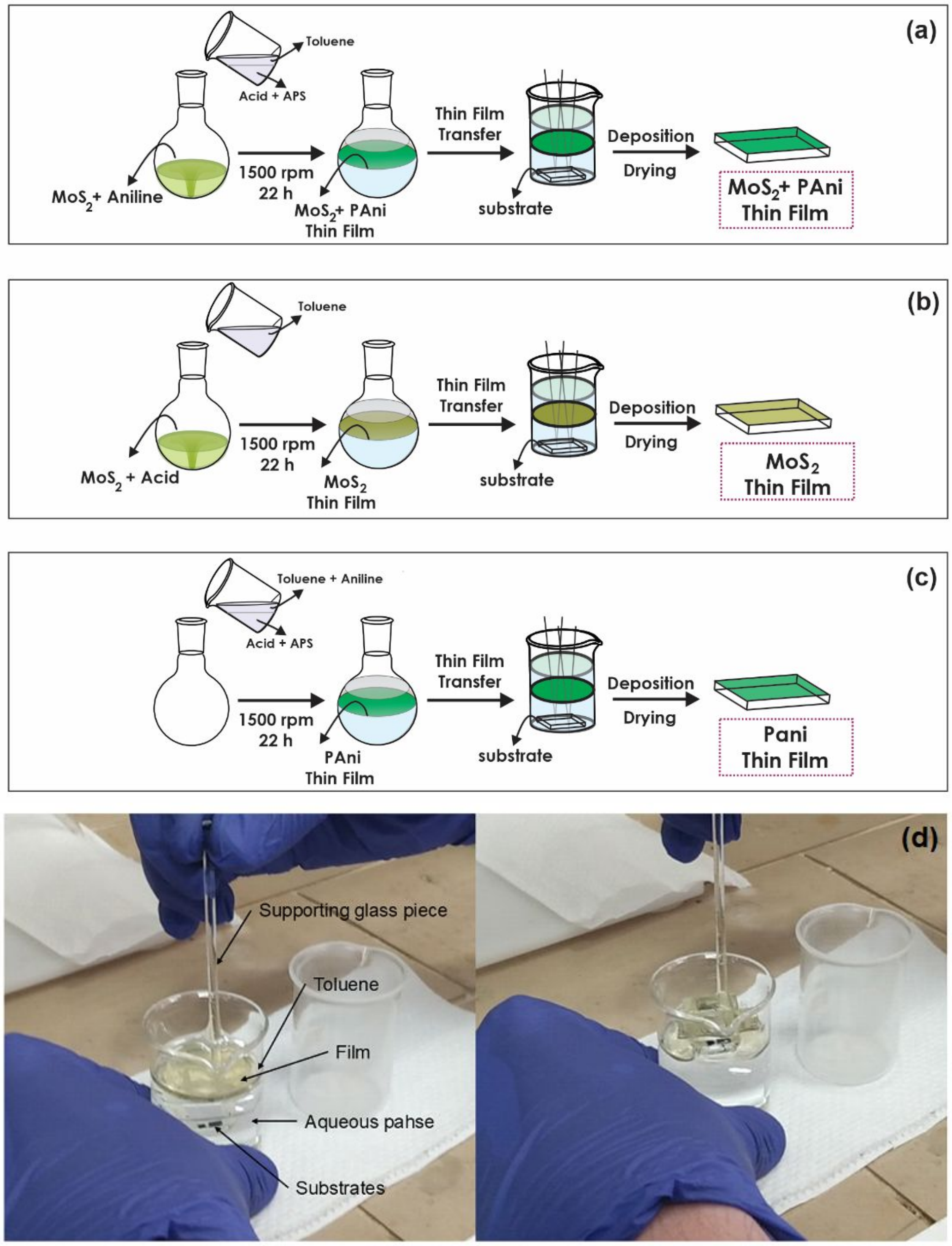

Fig. S1. Schematic representation of thin film synthesis and deposition of (a) MoS_2/PAni; (b) neat MoS_2 and (c) neat PAni; (d) picture showing the steps of the thin film deposition process.

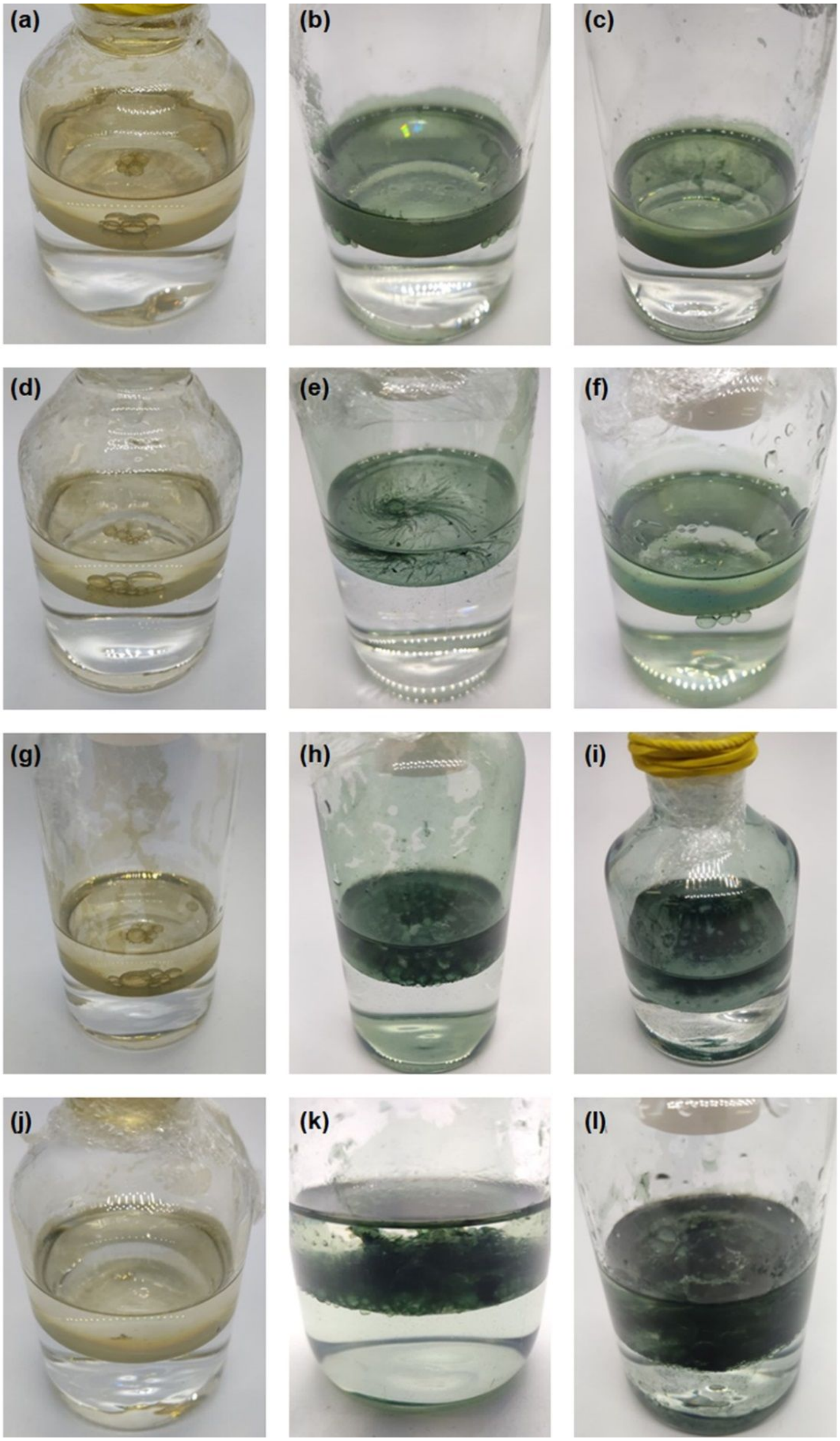

Fig. S2. Digital images of (a) M-S0; (b) P-S0; (c) MP-S0; (d) M-C0; (e) P-C0; (f) MP-C0; (g) M-S1; (h) P-S1; (i) MP-S1; (j) M-C1; (k) P-C1; and (l) MP-C1, at the liquid-liquid interface.

Table S1. Mean thickness of the films.

Sample	Thickness (nm)	Sample	Thickness (nm)	Sample	Thickness (nm)
**P-S0**	455 ± 133	**P-S1**	154 ± 26	**M-S0**	64 ± 8
**MP-S0**	379 ± 58	**MP-S1**	152 ± 32	**M-S1**	78 ± 18
**P-C0**	179 ± 24	**P-C1**	72 ±15	**M-C1**	52 ± 5
**MP-C0**	254 ± 58	**MP-C1**	61 ± 9	**M-C1**	95 ± 16

Table S2. Transmittance values at 550 nm of the films.

Sample	$T(\%)_{550\ nm}$	Sample	$T(\%)_{550\ nm}$
M-S0	62	P-C0	77
M-C0	35	MP-C0	60
M-S1	55	P-S1	76
M-C1	60	MP-S1	75
P-S0	39	P-C1	78
MP-S0	43	MP-C1	73

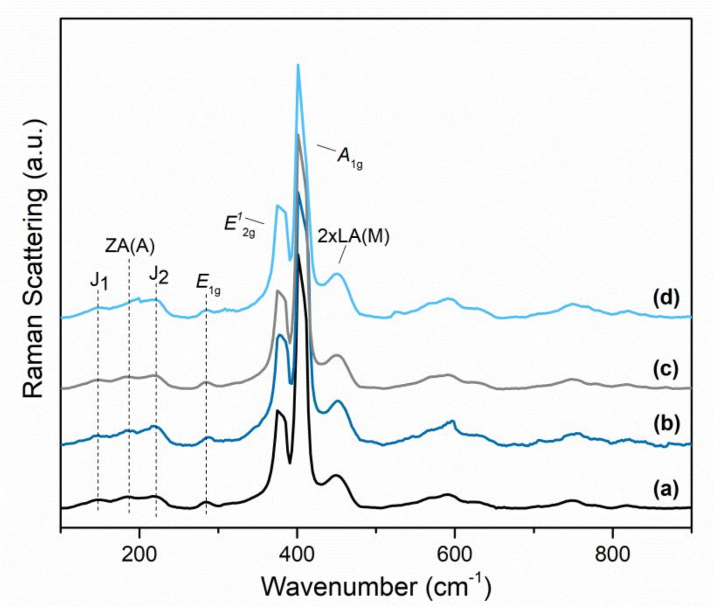

Fig. S3. Low frequency Raman spectra of (a) M-S0; (b) M-C0; (c) M-S1; and (d) M-C1.

Table S3 – Assignments to observed molybdenum oxides Raman bands.

Band Position (cm^{-1})	Attribution
569	MoO_2 - O-Mo streching
590	MoO_2 - O-Mo streching
600	MoO_3 - B_{1u}
662	MoO_3 - O-Mo-O stretching
749	MoO_2 - O-Mo streching
820	MoO_3 - Mo=O

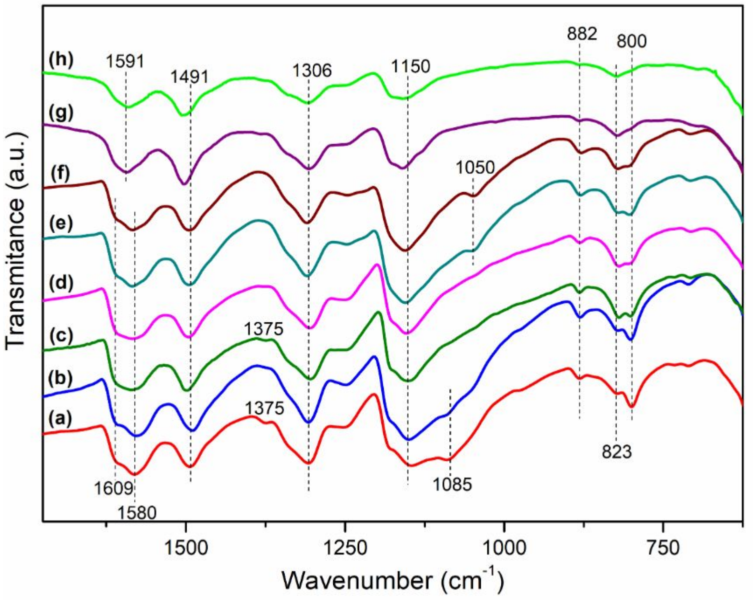

Fig. S4. FT-IR spectra of (a) P-S0; (b) MP-S0; (c) P-C0; (d) MP-C0; (e) P-S1; (f) MP-S1; (g) P-C1; and (h) MP-C1.

FT-IR discussion

Firstly, the bands at 1580 and 1491 cm^{-1} are assigned to quinoid and benzenoid ring stretching, respectively. The ratio between the areas under these two bands can be utilized to estimate the oxidation state of PAni, where higher A_{1491}/A_{1580} relationship indicates a less oxidized polymer.[1] Analyzing these bands, it is noticeable that generally, the composites appear to have higher intensity for the band at 1491 cm^{-1}, while the other one appears to shrink or become less defined, corroborating the fact the MoS_2 favors the formation of less oxidized polyaniline. The effect of dopant and synthesis pH is also appreciable again,

following the same trends observed and discussed in Raman spectroscopy, with HCl doped films being more oxidized than H_2SO_4 doped ones, and films obtained at pH 1 being more oxidized than the ones synthesized at pH 0.

The bands at 1609, 1306 and 1150 cm^{-1} provide some insight into the doping level of the polymer. The first one is assigned to inactive *v*(C=C) stretching modes induced by the formation of polarons; the second is related to *v*(C~N) vibrations in delocalized polarons; and the one at 1150 cm^{-1} rises from vibration of the ($-NH^+=$) structure.[1,2] All three bands also appear to be relatively more intense and defined in the composites, comparing to their corresponding neat polymers, which again indicates that the presence of MoS_2 induces higher protonation and doping of the polymer. Additionally, the weak band at 1375 cm^{-1}, that can only be observed in the neat polymers, is related to deprotonated portions of the polymer chains, further corroborating the higher degree of protonation in the composites.[3]

In the smaller frequency region, the last three bands identified provide conformational information about the polymer. The first one, at 882 cm^{-1} is assigned to C-H out-of-plane deformations, while the other two, at 823 and 800 cm^{-1} are related to C-H and 1,4 substituted rings out-of-plane deformations, respectively.[4] The higher relative intensity of the first, combined with the attenuated intensities of the last two, in the composites, supports the information that the MoS_2 stabilizes a more planar conformation of polyaniline. Lastly, the bands at 1050-1085 cm^{-1} can be assigned to *v*(S=O) stretching in the sulfate anions, which is why they can only be observed in the H_2SO_4 doped films.[4]

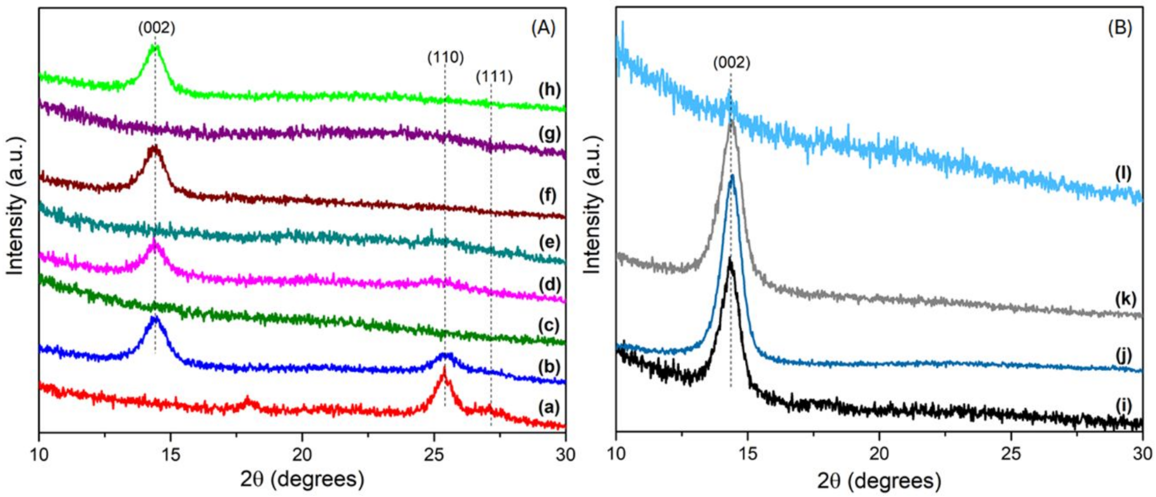

Fig. S5. XRD profiles of (a) P-S0; (b) MP-S0; (c) P-C0; (d) MP-C0; (e) P-S1; (f) MP-S1; (g) P-C1; (h) MP-C1; (i) M-S0; (j) M-C0; (k) M-S1; and (l) M-C1.

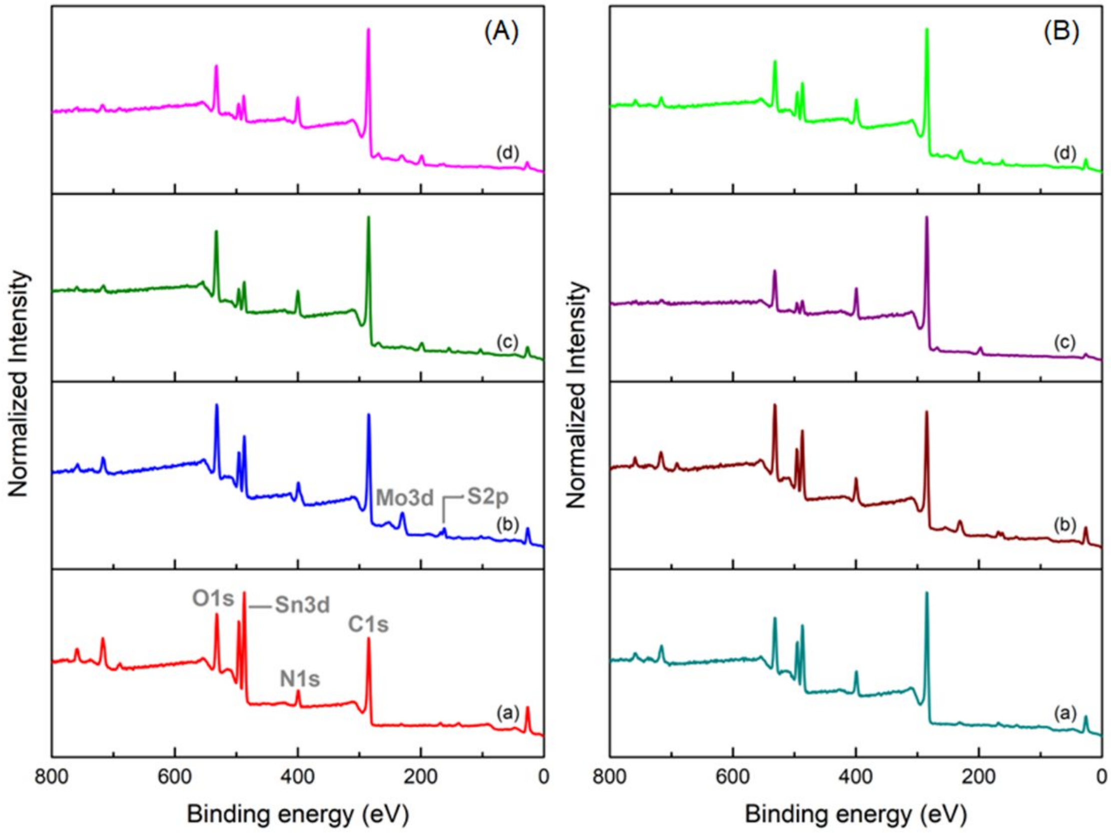

Fig. S6. XPS survey spectra of (A) (a) P-S0; (b) MP-S0; (c) P-C0; (d) MP-C0; and (B) (a) P-S1; (b) MP-S1, (c) P-C1, and (d) MP-C1 films.

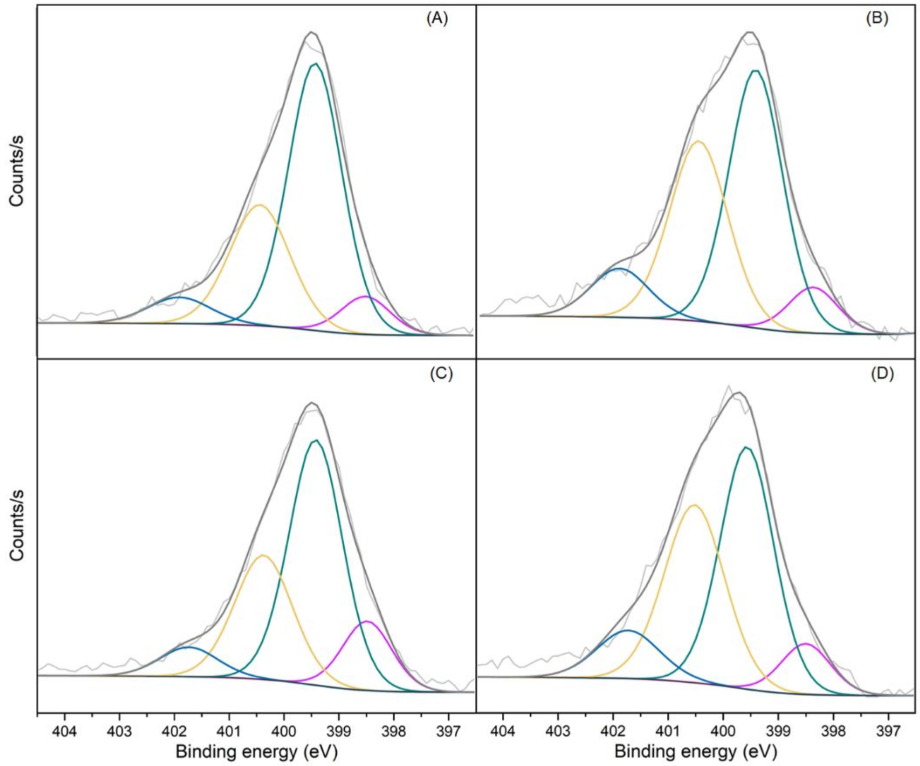

Fig. S7. N1s core level spectra for (A) P-S0; (B) P-C0; (C) P-S1 and (D) P-C1films.

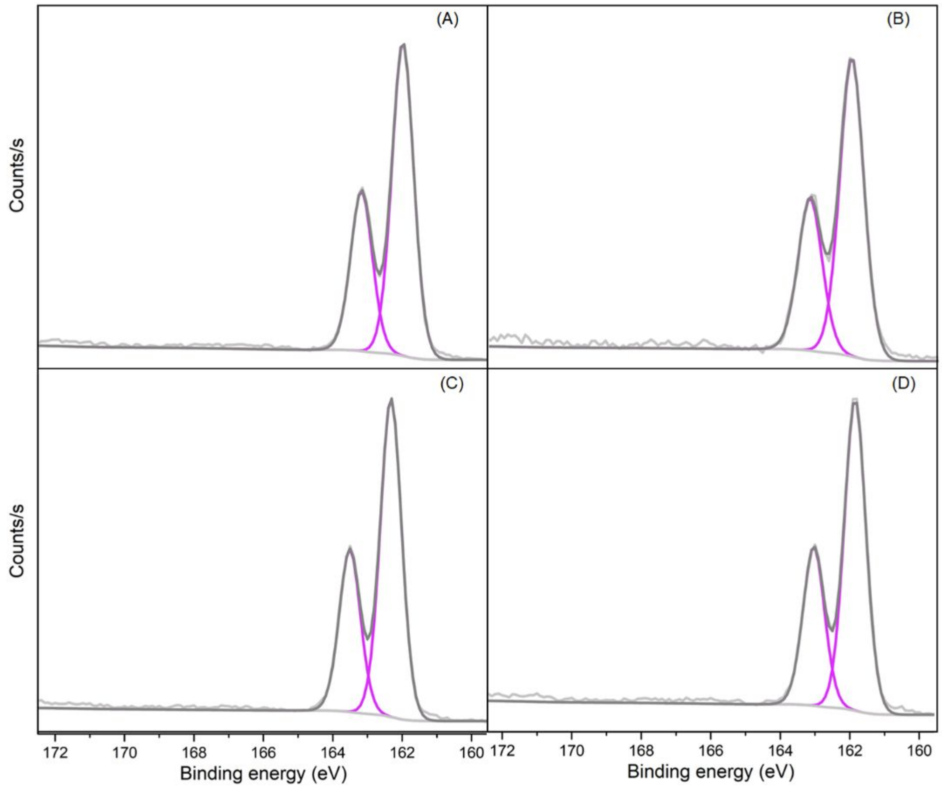

Fig. S8. S2p core level spectra for (A) M-S0; (B) M-C0; (C) M-S1 and (D) M-C1films.

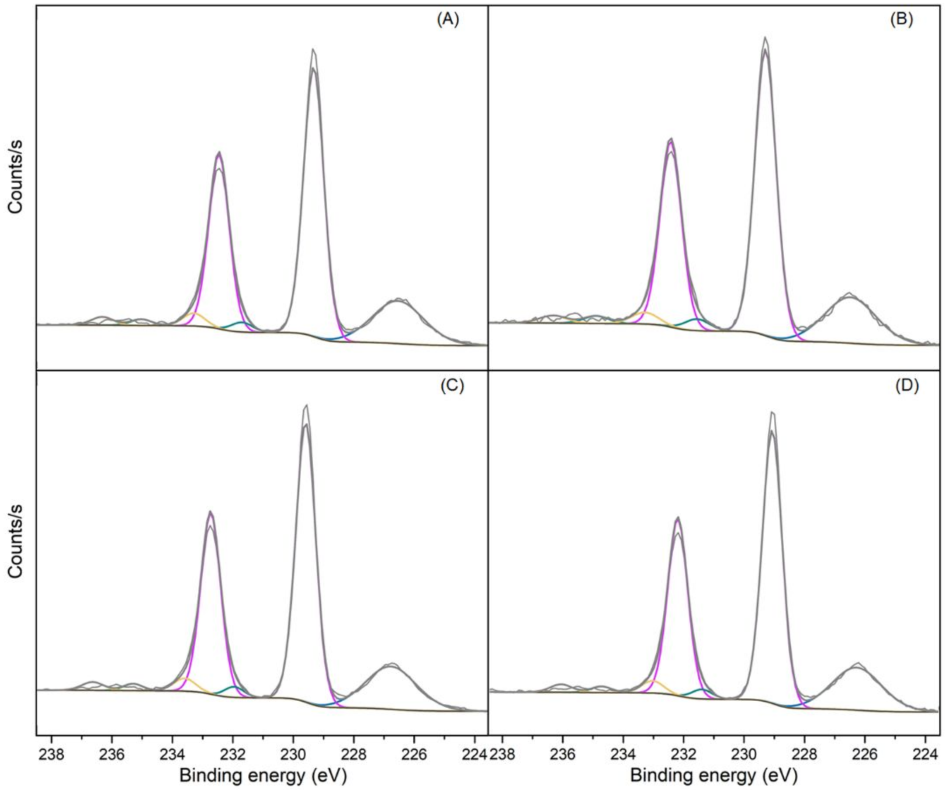

Figure S9. Mo3d core level spectra for (A) M-S0; (B) M-C0; (C) M-S1 and (D) M-C1films.

Table S4. Volumetric capacitances for PAni and MoS_2 based materials reported in the literature and in this work.

**Material**	**Volumetric Capacitance ($F\ cm^{-3}$)**	**Reference**
PAni nanofibers	93	[5]
PEDOT/MoS_2	174	[6]
SWNT/PAni	184	[7]
PEDOT/rGO/MoS_2	338	[6]
PAni nanowires	588	[8]
Stacked PAni/graphene	736	[9]
MX/PAni NPs	873	[10]
MoS_2/PAni	164 – 846	This work

(PEDOT = poly(3 4-ethylenedioxythiophene); SWNT = single wall carbon nanotube; rGO = reduced graphene oxide; MX = MXene ($Ti_3C_2T_x$); NPs = nanoparticles.

REFERENCES

(1) Furukawa, Y.; Ueda, F.; Hyodo, Y.; Harada, I.; Nakajima, T.; Kawagoe, T. Vibrational Spectra and Structure of Polyaniline. *Macromolecules* **1988**, *21* (5), 1297–1305. https://doi.org/10.1021/ma00183a020.

(2) Sariciftci, N. S.; Bartonek, M.; Kuzmany, H.; Neugebauer, H.; Neckel, A. Analysis of Various Doping Mechanisms in Polyaniline by Optical, FTIR and Raman Spectroscopy. *Synth Met* **1989**, *29* (1), 193–202. https://doi.org/10.1016/0379-6779(89)90296-8.

(3) Kaiser, A. B. Electronic Transport Properties of Conducting Polymers and Carbon Nanotubes. *Reports on Progress in Physics* **2001**, *64* (1), 1–49. https://doi.org/10.1088/0034-4885/64/1/201.

(4) Trchová, M.; Stejskal, J. Polyaniline: The Infrared Spectroscopy of Conducting Polymer Nanotubes (IUPAC Technical Report). *Pure and Applied Chemistry* **2011**, *83* (10), 1803–1817. https://doi.org/10.1351/PAC-REP-10-02-01.

(5) Maeng, J.; Kim, Y.-J.; Meng, C.; Irazoqui, P. P. Three-Dimensional Microcavity Array Electrodes for High-Capacitance All-Solid-State Flexible Microsupercapacitors. *ACS Appl Mater Interfaces* **2016**, *8* (21), 13458–13465. https://doi.org/10.1021/acsami.6b03559.

(6) Zhou, Q.; Lv, G.; Wang, X.; Teng, W.; Hu, P.; Du, Y.; Li, H.; Hu, Y.; Liu, W.; Wang, J. Constructing a Hierarchical Ternary Hybrid of PEDOT:PSS/rGO/MoS_2 as an Efficient Electrode for a Flexible Fiber-Shaped Supercapacitor. *ACS Appl Energy Mater* **2023**, *6* (11), 5797–5805. https://doi.org/10.1021/acsaem.3c00187.

(7) de Souza, V. H. R.; Oliveira, M. M.; Zarbin, A. J. G. Thin and Flexible All-Solid Supercapacitor Prepared from Novel Single Wall Carbon Nanotubes/Polyaniline Thin Films Obtained in Liquid–Liquid Interfaces. *J Power Sources* **2014**, *260*, 34–42. https://doi.org/10.1016/j.jpowsour.2014.02.070.

(8) Wang, K.; Zou, W.; Quan, B.; Yu, A.; Wu, H.; Jiang, P.; Wei, Z. An All-Solid-State Flexible Micro-supercapacitor on a Chip. *Adv Energy Mater* **2011**, *1* (6), 1068–1072. https://doi.org/10.1002/aenm.201100488.

(9) Wu, Z.; Parvez, K.; Li, S.; Yang, S.; Liu, Z.; Liu, S.; Feng, X.; Müllen, K. Alternating Stacked Graphene-Conducting Polymer Compact Films with Ultrahigh Areal and Volumetric Capacitances for High-Energy Micro-Supercapacitors. *Advanced Materials* **2015**, *27* (27), 4054–4061. https://doi.org/10.1002/adma.201501643.

(10) Wang, X.; Wang, Y.; Liu, D.; Li, X.; Xiao, H.; Ma, Y.; Xu, M.; Yuan, G.; Chen, G. Opening MXene Ion Transport Channels by Intercalating PANI Nanoparticles from the Self-Assembly Approach for High Volumetric and Areal

Energy Density Supercapacitors. *ACS Appl Mater Interfaces* 2021, *13* (26), 30633–30642. https://doi.org/10.1021/acsami.1c06934.
